# Validation of an instrument for the evaluation of exchange transfusion (INEXTUS) via an OSCE

**DOI:** 10.1186/s12909-022-03546-w

**Published:** 2022-06-20

**Authors:** María José Maldonado Calderón, Sergio Iván Agudelo Pérez, Natalia Becerra, Juan David Suarez

**Affiliations:** 1 Universidad de La Sabana and Clínica Universidad de La Sabana, Chía, Colombia; 2 School of Medicine, Universidad de La Sabana, Chía, Colombia; 3 School of Medicine, Universidad de La Sabana, Chía, Colombia; 4 School of Medicine, Resident of Paediatrics, Universidad de La Sabana, Chía, Colombia

**Keywords:** Hyperbilirubinemia, Exchange transfusion, Education, OSCE

## Abstract

**Introduction:**

Exchange transfusion is the treatment of choice for patients with severe hyperbilirubinemia who do not respond to phototherapy. This procedure is highly complex and requires substantial expertise to perform, however it´s not done frequently enough to guarantee adequate training. Traditional learning scenarios do not have a space reserved for teaching this procedure or an instrument that fully and objectively evaluates the skills that a professional must acquire.

**Objectives:**

The purpose was to construct and evaluate the INEXTUS instrument´s validity evidence relevant to internal structure, in a simulated scenario through the performance of an objective structured clinical exam (OSCE).

**Materials and methods:**

The Delphi consensus methodology was utilized to design the instrument; six experts participated through three rounds using the Google Forms platform. The categories and items previously obtained were subjected to validation by nine experts through a dichotomous survey. Prior to data collection, the evaluators were trained through a pilot test with 10 medical students. Subsequently, all residents of a paediatric programme were evaluated through the OSCE methodology in a simulated scenario, with 6 stations, of a clinical case of a new-born with an explicit need for exchange transfusion. During their participation in the scenario, the residents were first evaluated with the instrument developed. Additionally, audio and video filming of all students who participated was performed with the aim of conducting a second evaluation two weeks after the first four evaluators participated.

**Results:**

The final INEXTUS instrument consists of 46 subitems grouped into 23 items divided into 6 categories, demonstrating an inter-rater intraclass correlation coefficient of 0.96 (95% CI 0.94, 0.98 *p*-value < 0.001). For the Fleiss Kappa of the 23 items evaluated, concordance was evaluated for 14 items but could not be determined for the 9 remaining items because all the ratings were equal, either because the items were not performed or they were all performed adequately. Of the 14 items, 9 good scores were obtained (95% CI 0.61 to 0.8; *p* value < 0.001), and 5 very good scores were obtained (95% CI 0.81 to 1; *p* value < 0.001).

**Conclusions:**

The INEXTUS instrument evaluates exchange transfusion skills in medical personnel in training in simulated scenarios using the OSCE methodology; it has high validity and reliability and is a high-impact educational tool.

## Introduction

Jaundice is the leading cause of hospitalization in neonatal intensive care units (NICU) [1]. Patients who present severe hyperbilirubinemia and do not respond to initial management with phototherapy have a high risk of bilirubin-induced neurotoxicity, requiring immediate management by exchange transfusion, a highly complex procedure that leads to adverse events in 67–87% of cases, depending on the expertise of the professional and the gestational age of the patient [2].

Exchange transfusion consists of replacing the neonatal blood volume (recommended that it be a double volume). The goal is to lower serum bilirubin levels and remove circulating maternal antibodies from circulation. The choice of donor blood type depends on the hemoclassification of the mother and the neonate. There are different routes for carrying out the procedure. To carry out the procedure, it must be considered to connect sequentially to the line: catheter for drainage of extracted blood, that of donor blood and a syringe pre-filled with saline solution. The exchange volume of each aliquot should be 5–10% of the infant's blood volume [3, 4].

The advent of new methods to prevent jaundice from Rh incompatibility, such as the administration of anti-D immunoglobulin and the development of advanced noninvasive technology for the treatment of jaundice such as phototherapy, has led to a decrease in the need for exchange transfusion, especially in developed countries, where exchange transfusion rates are close to 1.9 per 100,000 live births [5, 6]. Second, there has been a lack of competence in the skills of professionals to perform this procedure, given the limited opportunities during postgraduate education and later in paediatric practice, which leads to an increase in adverse effects such as infection, cardiac arrest and death [6].

Clinical learning scenarios do not have a space reserved for teaching this procedure or a method that fully and objectively evaluates the skills that the professional must acquire. The Objective Structured Clinical Examination (OSCE) arises as a methodology aimed at evaluating in an objective, planned and structured way the components that make up clinical skill in a simulated scenario [7]. The OSCE is presented as a valid and reliable instrument for the evaluation of clinical components of the medical profession, for which it is necessary to appropriately design both the test and the instrument that evaluates the acquired skills [8–11].

The instrument ensures that the acquisition of skills is evaluated objectively, avoiding biases or impartial and unfounded judgements [12, 13]. An evaluative instrument can be useful to score clinicians frequently and guarantee the quality of the procedure, compare the skills between doctors, ascertain retentive skills and provide feedback during training [12].

Both a simulated scenario and an instrument that evaluates the performance of the student in itself is especially relevant in clinical practice scenarios that occur sporadically and in those of high complexity [14]. The exchange transfusion procedure is high-complexity procedure, and despite being performed infrequently, if indicated, it changes the neurological outcome and even prevents the death of patients severely compromised by hyperbilirubinemia [15, 16]. Despite the existence of multiple management guidelines in which the exchange transfusion technique is systematically explained in detail, after conducting a literature search on the subject, an instrument validated to measure paediatric residents´neonatal transfusion skills is not available.

The objective of the present study is to construct and evaluate validity evidence relevant to internal structure an instrument that evaluates skills in exchange transfusion and to evaluate its validity and reliability in a simulated scenario through an OSCE. The rubric designed will support the development of the research protocol published in Trials Maldonado et al. Trials (2020) 21:387. https://doi.org/10.1186/s13063-020-04312-3 and registered in clinical trials 18/08/2019 (**Trial registration:** NCT04070066).

## Methodology

### Development of INEXTUS

#### Phase 1: Bibliographic search and definition of need for designing an instrument

**Fig. 1:**
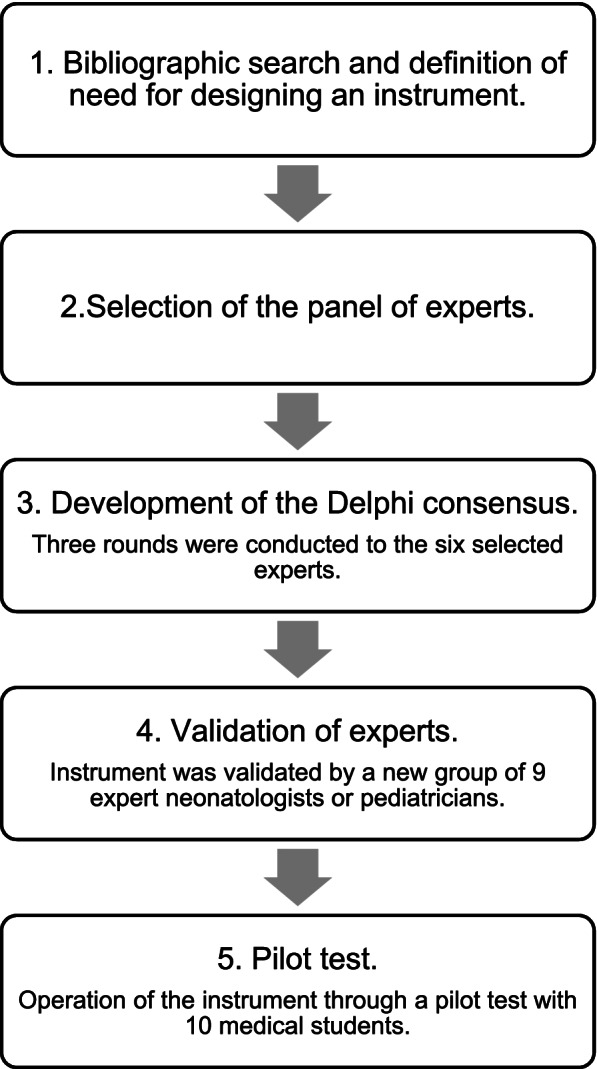
Flowchart Inextus development phases

The study began with an extensive literature review on the evaluation instruments of the exchange transfusion procedure in Medline, Scopus, Cochrane, LILACS and SciELO, determining that to date, there is no related instrument. The need for designing an exchange transfusion evaluation instrument (INEXTUS) is defined using the Delphi-type consensus methodology of multiple, individual rounds and without contact between the experts consulted. Figure 1 shows the Flowchart Intexus development Phases.

#### Phase 2: Selection of the panel of experts

A first preselection of 30 expert neonatologists or paediatricians came from different hospitals, with care experience in medium- and high-complexity neonatal units was proposed, selecting 12 who practiced in university hospitals with teaching experience and who had performed the exchange transfusion procedure during their professional practice. The pre-selected experts were invited to participate, and 6 of them agreed to participate.

#### Phase 3: Development of the Delphi consensus

Three rounds were conducted. First, a survey was sent to each expert via Google Forms®. The authors used an open-ended question to instruct the experts to list the skills for exchange transfusion. Once the results of the first round were in, the authors compiled and categorized the skills, constructing a general matrix from which the second round was carried out. In this questionnaire, all items were listed with questions on a 5-point Likert scale and to include an item definitively in the instrument, a cut-off point of 80% or more was established for *totally agree* and *agree*; and those items with 80% or more for *totally disagree* or *disagree* were excluded. After this round, a total of 25 items were excluded, obtaining a preliminary instrument with 3 categories, 18 items and 20 subitems. In the third round, the research group submitted the questionnaire again to the experts for their evaluation, asking their agreement on including each item through a dichotomous survey (yes or no). In this round, only one item was excluded.

#### Phase 4: Validation of experts

A new group of 9 expert neonatologists or paediatricians was contacted to send them the instrument for their consideration of whether they agreed with each of the items included. A survey was sent via Google Forms® with questions on a dichotomous scale of Yes or No responses. In this survey, they could make additional comments where they could suggest including an item or reorganizing the categories and related items. After this expert validation, an instrument of 6 categories, 23 items and 46 subitems was obtained.

#### Phase 5: Pilot test

Prior to data collection, the evaluators were trained in the operation of the instrument through a pilot test with 10 final year medical students so the evaluator could assess their comprehension of the instrument and the feasibility of applying it.

##### Population, procedures and scenario

The study population corresponds to residents enrolled in the paediatrics programme of the Universidad de La Sabana. It was developed in the Simulated Hospital of the Universidad de La Sabana during the month of January 2020. The Simulated Hospital is an academic and research centre that teaches clinical simulation with high-fidelity simulators in simulated clinical scenarios. The pediatric residents included in the study received training prior to the start of data collection. For this, an educational strategy was developed that consisted of a video and guide of the procedure and recommended references for individual reading. Students who participated in the study signed an informed consent, and it was clarified that their participation in the study would not affect their academic situation and that the confidentiality of the data would be guaranteed.

All 24 residents underwent the evaluation and application of the checklist for a standardized clinical case according to the OSCE methodology.

The clinical case scenario was a term neonate with normal weight for gestational age of 13 h of life, blood group incompatibility, clinical jaundice, haemolysis and signs of hyperbilirubinemic encephalopathy who was with the mother.

**Fig. 2:**
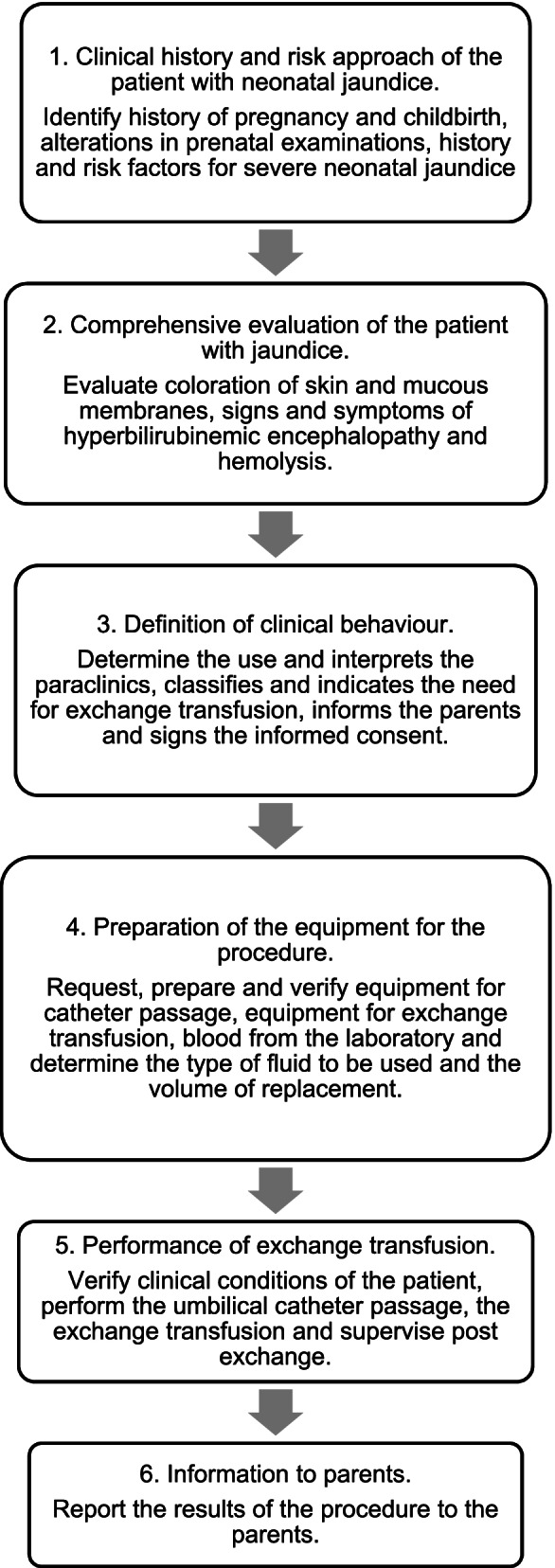
Flowchart OSCE stations

**Fig. 3:**
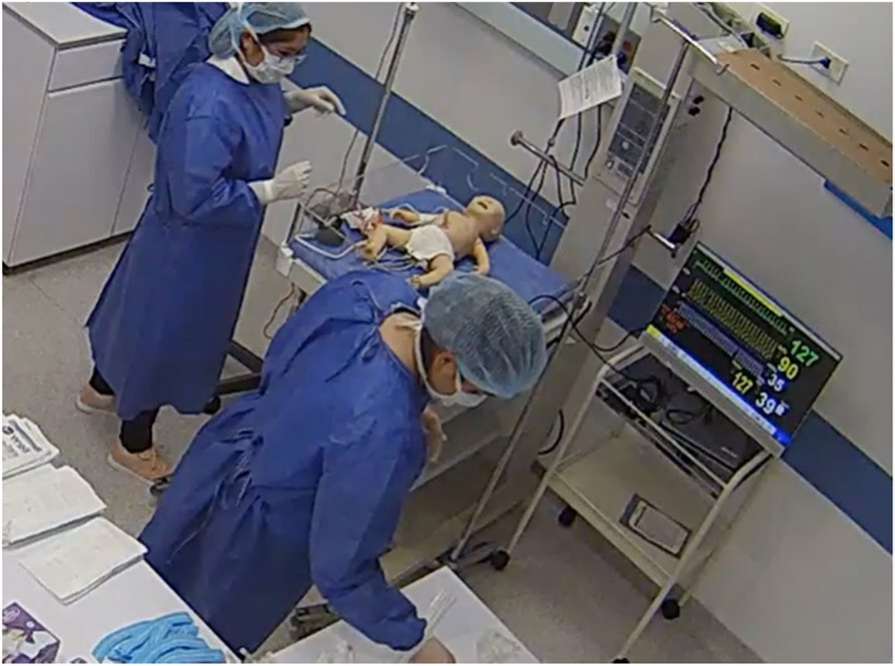
simulated scenario

This evaluation scenario consisted of six stations, (Fig. 2: Flow chart OSCE stations) that evaluated each subtopic of the evaluation instrument. Patients (simulated mother) participated in stations one and two, head nurses in station five, and high-fidelity neonatal simulators with monitors and equipment for exchange transfusion. Figure 3 shows the simulated scenario.

The evaluators responsible for applying the instrument were professors from the School of Medicine and the Universidad de La Sabana Clinic, two neonatologists and two paediatricians, with experience in the procedure and in medical education in simulated scenarios.

During the participation of the paediatric residents in the scenario, the first assessment was carried out by the evaluators using the instrument developed (INEXTUS—Exchange transfusion evaluation instrument, Universidad de La Sabana). In order to perform the proof reproof intra-raters reliability, audio and video filming of the entire participation of the student in the scenario was made with the objective of performing the second assessment two weeks after the four evaluators first participated.

##### Statistical analysis

A sample size was calculated for inter-rater and intra-rater reliability, taking into account an expected intraclass correlation coefficient (ICC) of 0.8, with a 95% confidence interval (CI) of 0.2 and alpha error of 0.05, power of 80% and with four observers a total of 24 participants [17].

Each item of the instrument was scored dichotomously: 0 (did not meet or partially met) and 1 (all met). The score of each subtopic was obtained by dividing the student’s score by the maximum possible and multiplying it by 100. Similarly, the total score was obtained from the score of each subtopic divided by the possible total and multiplied by 100. For inter-observer and intra-observer reliability, the ICC was calculated with two mixed factors and single measures with the 95% CI for the percentage of the total score of the instrument. Likewise, to evaluate whether agreement of the classification was partially met and/or not met (0) and all met (1) for the inter- and intra-evaluator test–retest, the Fleiss Kappa coefficient with 95% CI and the Kappa coefficient 95% CI were calculated, respectively.

The recording and analysis of the information was performed in Microsoft Excel for Windows 10 and IBM SPSS Statistics 26 software. A value of *p* < *0.05* was established as a value of significance.

## Results

Twenty-four residents of the paediatrics programme participated, 6 of whom had completed their studies and not yet graduated. The sociodemographic characteristics are summarized in Table 1. The four evaluators responsible for applying the instrument were professors from the School of Medicine and the Universidad de La Sabana Clinic (two neonatologists and two paediatricians), with experience in the procedure and in medical education in simulated scenarios (Table 2).

**Table 1:** Characteristics of the participants

Characteristic		n	%
**Gender**	Male	2	8
	Female	22	92
**Age**	25–30 years	19	79
	31–35 years	5	21
**Year of residence**	First	6	25
	Second	6	25
	Third	6	25
	Awaiting degree	6	25

**Table 2:** Residents rating (%) for each station

Evaluators	Station 1. Clinical history and risk approach of the patient with neonatal jaundice	Station 2. Comprehensive evaluation of the patient with jaundice	Station 3: Definition of clinical behaviour	Station 4. Preparation of the equipment for the procedure	Station 5. Performing exchange transfusion	Station 6. Information to parents
1	76,9	65,5	85,0	48,8	23,3	83,3
2	75,3	64,3	85,4	45,4	22,8	75,0
3	76,0	66,1	81,3	47,1	23,6	66,7
4	74,7	64,3	80,0	47,5	25,8	66,7

### Assessment instrument

A total of 9 neonatologists with academic and healthcare experience participated in the consensus rounds. One was head of department in their School of Medicine, and four were coordinators or heads of neonatal units. All exercised their clinical practice in university hospitals, responsible for training paediatric residents.

The final instrument incorporated the different aspects of the exchange transfusion procedure, from evaluating and identifying patients who require the procedure, to patient preparation and stabilization, requesting tests and necessary equipment, to managing the parents and the technique of the procedure.

The final instrument to assess skills in exchange transfusion (INEXTUS) consists of 46 subitems grouped into 23 items, divided into 6 categories: Clinical history and risk approach of patients with neonatal jaundice, Comprehensive assessment of the patient with jaundice, Definition of clinical behaviour, Preparing equipment for the procedure, Perform the exchange transfusion, Inform and manage the parents. In the pilot test, the feasibility of the application of the instrument and its easy and rapid comprehension by the evaluator was observed (see Table 3 INEXTUS Instrument (Instrument of exchange transfusion of La Sabana)).

**Table 3:** Final INEXTUS instrument

INEXTUS INSTRUMENT FOR TRAINING AND EVALUATION OF EXCHANGE TRANSFUSION		
**Station 1. Clinical history and risk approach of the patient with neonatal jaundice**	**Yes**	**No**
1. The student identifies background of pregnancy and childbirth		
a. Inquires about obstetric care and birth spacing		
b. Asks about history of the evolution of previous pregnancies (emphasis on abortions, deaths or stillbirths)		
c. Asks about pathological history during pregnancy (UTI, infections, preeclampsia, tropical exanthematous diseases)		
d. Inquires about the number of prenatal check-ups and when they started		
e. Determines the gestational and chronological age of the patient		
2. Review prenatal exams (Complete blood count, serology, blood typing and indirect Coombs, thyroid stimulating hormone, ultrasound. Others according to particular case)		
3. The student identifies history and risk factors for severe neonatal jaundice		
a. Jaundice in the first 24 h		
b. Group incompatibility—verifies maternal and neonatal blood typing		
c. Coombs positive		
d. Gestational age from 35 to 37 weeks		
e. Family history of haemolytic disease		
f. Clinical factors: cephalohaematoma and exclusive breastfeeding		
g. History of maternal blood ingestion		
**Percentage of skills met at Station 1: _____%**		
**Station 2. Comprehensive evaluation of the patient with jaundice**	**Yes**	**No**
1. Evaluates skin and mucosal colouration describing percentage of extent of involvement		
2. Evaluates signs and symptoms of acute hyperbilirubinemic encephalopathy		
a. Hypotonia		
b. Lethargy		
c. Poor suction		
3. Evaluates signs and symptoms compatible with haemolysis		
a. Evaluates vital signs of the new-born		
b. Evaluates state of consciousness		
c. Performs abdominal palpation to rule out hepatomegaly		
**Percentage of skills met at Station 2: _____%**		
**Station 3: Definition of clinical behaviour**	**Yes**	**No**
1. The student determines the use of paraclinical tests and requests:		
a. Complete Blood Count. (CBC)		
b. Total and direct bilirubin levels		
c. Direct Coombs test of the new-born		
d. Reticulocytes		
e. Peripheral blood smear		
2. The student performs the comprehensive interpretation of the data and classifies the patient		
a. Classifies the type of hyperbilirubinemia according to the results as severe		
b. Orders immediate intensive phototherapy		
3. Indicates that exchange transfusion is required according to the use of the nomogram		
4. The student explains the behaviour to the parents and resolves their concerns		
5. Sign informed consent		
**Percentage of skills met at Station 3: _____%**		
**Station 4. Preparation of the equipment for the procedure**	**Yes**	**No**
1. The student verifies that he or she has the appropriate personnel to perform the procedure (head nurse and nursing assistant)		
2. Properly selects the catheter		
i. Preterm: 2.5f; Near-term: 5f; At term: 5-7f		
3. Requests, prepares and verifies equipment for catheter passage (Gloves, gauze, sterile gown and drapes, mask, cap, cloths, sterile gauze, scalpel blades, 3 and 4 zero floss, syringes loaded with heparinized serum, chlorhexidine solution, surgical team)	1	2
4. Request, prepare and verify equipment for exchange transfusion		
i. 5,10 and 20 ml Syringes		
ii. Three-way stopcocks		
iii. Bag to dispose of blood and metal support to hang it		
iv. Pipes to connect the systems		
5. The student requests the blood from the laboratory and verifies it when it is delivered		
i. Properly requests blood according to group and Rh compatibility		
ii. Request fresh blood less than 5 days old		
6.The student determines the type of fluid to be used and the volume of replacement		
i. The student performs the adequate calculation of the replacement volume considering the diagnosis of the patient (5 cc in premature infants, 10 cc at term)		
**Percentage of skills met at Station 4: _____%**		
**Station 5. Performance of exchange transfusion**	**Yes**	**No**
1. The student verifies the conditions of the patient and monitors vital signs		
i. Check blood pressure		
ii. Heart rate		
iii. Respiratory rate		
iv. Oxygen saturation		
2. The student performs the umbilical catheter passage appropriately		
i. Verify the distance at which the catheter will be inserted		
ii. Follow the rules of asepsis and antisepsis for performing the procedure		
iii. Adequately and successfully introduces the catheter to the previously determined site		
iv. Check its permeability		
v. Fix the catheter		
vi. The student requests images to verify the position of the catheter (Request an abdominal x -ray)		
3. The student performs the exchange transfusion sequentially and in an orderly manner		
i. Properly connect the exchange transfusion system		
ii. Complies with the order of introducing the aliquot—blood extraction in the stipulated time and volume		
iii. Instructs the help staff to record in detail the meticulous aliquot process		
4. The student orders post-exchange transfusion monitoring to detect the appearance of early complications		
5. The student requests diagnostic testing at the end of the procedure and 6 h after it (blood count, bilirubins and serum electrolytes)		
**Percentage of skills met at Station 5: _____%**		
**Station 6. Information to parents**	**Yes**	**No**
1. The student reports the results of the procedure to the parents		
**Percentage of skills met at Station 6: _____%**		

### Reliability

The inter-rater intraclass correlation coefficient was 0.96 (95% CI 0.94, 0.98 *p* value < 0.001). The test–retest intra-rater reliability is shown in Table 4, obtaining excellent reliability values.

**Table 4:** Intra-rater test–retest reliability

	Intraclass correlation coefficient	95% CI		*p value*
Observer 1	0.99	0.98	0.99	*p* < 0.001
Observer 2	0.97	0.95	0.99	*p* < 0.001
Observer 3	0.96	0.92	0.98	*p* < 0.001
Observer 4	0.99	0.98	0.97	*p* < 0.001

Table 5 shows the Fleiss kappa coefficient for the agreement between evaluators of each item at each evaluated station. Of the 23 items, agreement for 14 items was possible to calculate since in the remaining 9, the assessments by the four evaluators was equal (evaluators assigned an equal rating to all the residents,either complied or not complied). Table 6 shows the intra-rater agreement (evaluator 1) test–retest for each item at each station, finding moderate to high agreement for 14 of the 23 items evaluated.

**Table 5:** Fleiss Kappa coefficient for each station item (inter-rater reliability)

Station	Fleiss’ kappa	95% CI	*p* value
item			
Station 1. Clinical history and risk approach of the patient with neonatal jaundice			
Identifies background of pregnancy and childbirth	0.75	(0.59, 0.91)	< 0.001
Reviews prenatal exams	0.67	(0.50, 0.83)	< 0.001
Identifies history and risk factors for severe neonatal jaundice^a^	-	-	-
Station 2. Comprehensive evaluation of the patient with jaundice			
Evaluates skin and mucosal colouration describing percentage of extent of involvement^a^	-	-	-
Evaluates signs and symptoms of acute hyperbilirubinemic encephalopathy	0.61	(0.45, 0.78)	< 0.001
Evaluates signs and symptoms compatible with haemolysis	0.84	(0.68, 1.00)	< 0.001
Station 3: Definition of clinical behaviour			
Determines whether to use diagnostic testing and requests the appropriate ones	0.75	(0.59, 0.92)	< 0.001
Performs comprehensive interpretation of the data and classifies the patient	0.60	(0.44, 0.76)	< 0.001
Indicates that exchange transfusion is required according to the use of the nomogram^a^	-	-	-
Explains the behaviour to the parents and resolves their concerns	0.78	(0.62, 0.95)	< 0.001
Signed informed consent	0.69	(0.52, 0.85)	< 0.001
Station 4. Preparation of the equipment for the procedure			
The student verifies that they have the appropriate personnel to perform the procedure	1.00	(0.83, 1.1)	< 0.001
Properly select the catheter^a^	-	-	-
Requests, prepares and verifies equipment for catheter passage	1.00	(0.83, 1.1)	< 0.001
Requests, prepares and checks equipment for exchange transfusion	-	-	-
Requests the blood from the laboratory and verifies it when it is delivered	0.88	(0.79, 1.04)	< 0.001
Determines the type of fluid to be used and the volume of replacement^a^	-	-	-
Station 5. Performing exchange transfusion			
Verifies patient condition and monitors vital signs^a^	-	-	-
Performs the passage of the umbilical catheter properly^a^	-	-	-
Performs sequential and orderly exchange transfusion^a^	-	-	-
Indicates post-exchange transfusion monitoring to detect the appearance of early complications^a^	1.00	(0.83, 1.1)	< 0.001
Requests diagnostic testing at the end of the procedure and 6 h after it^a^	0.70	(0.53, 0.86)	< 0.001
Station 6. Information to parents			
Reports the outcomes of the procedure to parents^a^	0.75	(0.59, 0.91)	< 0.001

Interpretation of the kappa values: < 0.2 poor, 0.21—0.40 weak, 0.41—0.60 moderate, 0.61—0.80 good, 0.81—1.00 very good

^a^The Fleiss Kappa coefficient could not be determined in these items since all the ratings were equal, either because it was not performed or all were performed adequately

**Table 6:** Kappa coefficient for each station item (intra-rater reliability for evaluator 1)

Station	Kappa	95% CI	*p* value
item			
Station 1. Clinical history and risk approach of the patient with neonatal jaundice			
Identifies background of pregnancy and childbirth	0.88	(0.48, 1.2)	< 0.001
Reviews prenatal exams	1.00	(0.60, 1.4)	< 0.001
Identifies history and risk factors for severe neonatal jaundice^a^	-	-	-
Station 2. Comprehensive evaluation of the patient with jaundice			
Evaluates skin and mucosal colouration describing percentage of extent of involvement^a^	-	-	-
Evaluates signs and symptoms of acute hyperbilirubinemic encephalopathy	0.59	(0.19, 0.99)	< 0.001
Evaluates signs and symptoms compatible with haemolysis	0.64	(0.24, 1.00)	< 0.001
Station 3: Definition of clinical behaviour			
Determines whether to use diagnostic testing and requests the appropriate ones	1.00	(0.60, 1.40)	< 0.001
Performs comprehensive interpretation of the data and classifies the patient	1.00	(0.60, 1.40)	< 0.001
Indicates that exchange transfusion is required according to the use of the nomogram^a^	-	-	-
Explains the behaviour to the parents and resolves their concerns	1.00	(0.60, 1.40)	< 0.001
Signed informed consent	1.00	(0.60, 1.40)	< 0.001
Station 4. Preparation of the equipment for the procedure			
The student verifies that they have the appropriate personnel to perform the procedure	1.00	(0.60, 1.40)	< 0.001
Properly select the catheter^a^	-	-	-
Requests, prepares and verifies equipment for catheter passage	1.00	(0.60, 1.40)	< 0.001
Requests, prepares and checks equipment for exchange transfusion	-	-	-
Requests the blood from the laboratory and verifies it when it is delivered	1.00	(0.60, 1.40)	< 0.001
Determines the type of fluid to be used and the volume of replacement^a^	-	-	-
Station 5. Performing exchange transfusion			
Verifies patient condition and monitors vital signs^a^	-	-	-
Performs the passage of the umbilical catheter properly^a^	-	-	-
Performs sequential and orderly exchange transfusion^a^	-	-	-
Indicates post-exchange transfusion monitoring to detect the appearance of early complications^a^	1.00	(0.60, 1.40)	< 0.001
Requests diagnostic testing at the end of the procedure and 6 h after it^a^	1.00	(0.60, 1.40)	< 0.001
Station 6. Information to parents			
Reports the outcomes of the procedure to parents^a^	1.00	(0.60, 1.40)	< 0.001

Interpretation of the kappa values: < 0.2 poor, 0.21—0.40 weak, 0.41—0.60 moderate, 0.61—0.80 good, 0.81—1.00 very good

^a^The Fleiss Kappa coefficient could not be determined in these items since all the ratings were equal, either because it was not performed or all were performed adequately

## Discussion

Through this work, we constructed and reviewed targeted validity evidence of the INEXTUS instrument intended to evaluate the skills of residents in training in exchange transfusion under simulated scenarios using an OSCE for its high validity and reliability, thus promoting learning through the realistic recreation of scenarios [18, 19]. The reliability estimated by the intraclass correlation coefficient and by the Fleiss Kappa is found reasonably reliable and reproducible ranges for use in the training of paediatric graduate studies. Validating the rubric allowed us to refine the content and make decisions about the items to be included. These data are important as input for generating a controlled educational environment based on clinical simulation in the exchange transfusion procedure [20].

Opportunities in daily practice to develop skills in exchange transfusion are scarce, and paediatric professionals do not reach a sufficient level of competence to master this procedure in the clinical setting [21]. Clinical simulation scenarios are proposed as a valid, reliable and effective teaching option for skills acquisition in graduate-level medical personnel in training [22, 23].

Exchange transfusion is a highly complex procedure that is performed in neonatal intensive care units and under specific medical indications, so it requires a high level of expertise [24]. To date, there are no reports in the literature of the development of a rubric to assess the exchange transfusion procedure; therefore, the instrument developed (INEXTUS) will allow a solution to a known problem in the training of future paediatricians, that together with the simulated scenario is an appropriate strategy for the development and evaluation of these skills [25, 26].

The assessment of clinical skills is a key objective in the training of human talent in health; however, it is a challenge since knowledge, skills and attitudes must be evaluated simultaneously. A continuous and comprehensive assessment drives learning, recognizing that it is a complex process that requires a consistent methodology through valid and reliable instruments [27]. The rubric designed for exchange transfusion allows assessing theoretical skills, communication, procedural skills, among others, allowing teachers and students to identify the skills and abilities that should be reinforced in a more granular way, similar to what has been described by Smith and collaborators during the development of a rubric to evaluate students during assessing virtual patients [28].

The present study has some limitations. The research was limited to 2 sources of validity evidence- test content and internal structure. Although a sample size was calculated for the main outcome, the number of students included may be low, and only pediatric residents from one university were included. This limits the generalizability of the results to other settings. Similarly, all residents were evaluated without stratifying the level of training, which could impact the results. However, the objective was to evaluate the reliability of the instrument, observing the reproducibility of the results when applied by the same evaluator, by different evaluators and at different times.

In this context, the future use of INEXTUS is recommended at stratified training levels, with a greater number of participants and from different universities and even scenarios (it may be already trained personnel). As strengths of the present study, the development of an instrument with content constructed through an in-depth search of the literature and the contribution of experts in the exchange transfusion procedure is identified, which supports its valid content. There were no problems during the evaluation in the simulated scenario or during the data collection. Within the limitations, it was not possible to directly evaluate evidence relevant to relationships to other measures since there is no instrument with which to compare it.

## Conclusions

The exchange transfusion instrument is validated based on reliability and its construction to be used to evaluate students during the development of an of exchange transfusion OSCE. The results obtained show that the validity of the instrument and the intra- and inter-rater reliability were achieved.

This instrument will allow the evaluation of paediatric students when performing the exchange transfusion procedure, providing teachers with objective information about their performance. Likewise, when the evaluation is developed in an OSCE, students can be trained, and objective feedback can be given to the participants. 

## Data Availability

The datasets used and/or analysed during the current study are available from the corresponding author on reasonable request.

## Abbreviations

OSCE: Objective Structured Clinical Examination
APP: American Academy of Pediatrics

## References

[1] Castaño JJ, Gallego JA, Guevara J, Gonzalez GD, Meneses GA, Pabon JD, Salazar CF (2013). Caracterización de neonatos con diagnóstico de hiperbilirrubinemia indirecta en la unidad neonatal del S.E.S. hospital de Caldas (Manizales-Colombia 2009–2013).

[2] Ree IMC (2017). Neonatal management and outcome in alloimmune hemolytic disease. Expert Rev Hematology.

[3] Murki S, Kumar P (2011). Blood exchange transfusion for infants with severe neonatal hyperbilirubinemia. Semin Perinatol.

[4] Martínez de la Barrera LI (2017). Ictericia neonatal - hiperbilirrubinemia indirecta. Programa de educación continua en Pediatría. Sociedad Colombiana de Pediatría.

[5] Jain A, Malhotra S, Marwaha N, Kumar P, Sharma RR (2018). Severe ABO hemolytic disease of fetus and newborn requiring blood exchange transfusion. Asian J Transfus Sci.

[6] Patra K, Storfer-Isser A, Siner B, Moore J, Hack M (2004). Adverse events associated with neonatal exchange transfusion in the 1990s. J Pediatr.

[7] Romero S. ECOE : Evaluación Clínica Objetiva Estructurada. Medicina de Familia (And). 2002;2:127–32. https://www.um.es/c/document_library/get_file?uuid=9fa20d68-26f6-430c-8451-7842598bea17&groupId=115466.

[8] Utili F (2007). Simulación en el aprendizaje, practica y certificación de las competencias en medicina. ARS MEDICA Revista De Ciencias Médicas.

[9] Diseño de una prueba evaluativa de competencias para el laboratorio de simulación de enfermería. Rodríguez Higueras ETDX (Tesis Doctorals en Xarxa) (2014). http://hdl.handle.net/10803/133371.

[10] Harden RM, Stevenson M, Downie WW, Wilson GM (1975). Assessment of clinical competence using objective structured examination. BMJ.

[11] Brailovsky CA, Grand'Maison P (2000). Using Evidence to Improve Evaluation: A Comprehensive Psychometric Assessment of a SP-Based OSCE Licensing Examination. Adv Health Sci Educ Theory Pract.

[12] Bouwmeester RN, Binkhorst M, Yamada NK, Geurtzen R, van Heijst A, Halamek LP, Draaisma J, Hogeveen M (2019). Appraisal of a scoring instrument for training and testing neonatal intubation skills. Arch Dis Child Fetal Neonatal Ed.

[13] Diseño y validación de un instrumento para evaluar la competencia intubación orotraqueal en escenario simuladoCollante Padilla A, Montenegro Chávez J, Educación M (2015). http://hdl.handle.net/10584/7613.

[14] Martinez-González, A y Soto-Estrada, G. Examen clinico objetivo estructurado. ¿El reto a vencer para ejercer la medicina? Revista Digital Universitaria (RDU). 2018;19 núm 6 noviembre-diciembre. 10.22201/codeic.16076079e.2018.v19n6.a12.

[15] American Academy of Pediatrics Subcommittee on Hyperbilirubinemia (2004). Management of hyperbilirubinemia in the newborn infant 35 or more weeks of gestation. Pediatrics.

[16] Steiner LA, Bizzarro MJ, Ehrenkranz RA, Gallagher PG (2007). A decline in the frequency of neonatal exchange transfusions and its effect on exchange-related morbidity and mortality. Pediatrics.

[17] Machin D, Campbell MJ, Tan SB, Tan, SH. Observer agreement studies. In: Sample sizes for clinical, laboratory and epidemiology studies. 4th ed. Wiley Blackwell; 2018. p. 31. 10.1002/9781118874905.

[18] Martínez JM (2005). Los métodos de evaluación de la competencia profesional: la evaluación clínica objetivo estructurada (ECOE). Educación Médica.

[19] Gómez L, Dávalos LG, Rodríguez PF, Blanco E, Viera RV, Rocha IC (2019). La evaluación clínica objetiva estructurada desde el área de formación médica general. Investigación en educación médica.

[20] Ker T, Bradley P. Simulation in medical education. In: Understanding medical education. Chichester: John Wiley & Sons, Ltd; 2013. p. 175–92. 10.1002/9781118472361.ch13.

[21] Philip AGS (2003). Historical perspectives. The Rise and Fall of Exchange Transfusion. Neoreviews.

[22] Paige JT, Arora S, Fernandez G, Seymour N (2015). Debriefing 101: training faculty to promote learning in simulation-based training. Am J Surg.

[23] Mayville ML (2011). Debriefing: The Essential Step in Simulation. Newborn Infant Nurs Rev.

[24] Murki S, Kumar P (2011). Blood exchange transfusion for infants with severe neonatal hyperbilirubinemia. Semin Perinatol.

[25] Kassab M, Kenner C. Simulation and neonatal nursing education. Newborn Infant Nurs Rev. 2011;11(1):8–9. 10.1053/j.nainr.2010.12.006.

[26] Ruiz AI, Angol E, Guevara O. La simulación clínica y el aprendizaje virtual. Tecnologías complementarias para la educación médica. Revista de la Facultad de Medicina. 2009;57(1):67–79. http://www.scielo.org.co/scielo.php?script=sci_arttext&pid=S012000112009000100009&lng=en&tlng=es.

[27] Reynolds C, Fisher K, Fairbrother H. A novel standardized rubric for medical student emergency medicine oral presentations. Western Journal of Emergency Medicine: Integrating Emergency Care with Population Health. 2019;20(4.1). Retrieved from https://escholarship.org/uc/item/9sw6c48v.

[28] Smith S, Kogan JR, Berman NB, Dell MS, Brock DM, Robins LS (2016). The development and preliminary validation of a rubric to assess medical students' written summary statements in virtual patient cases. Acad Med.

